# Targeted transperineal biopsy of the prostate has limited additional benefit over background cores for larger MRI-identified tumors

**DOI:** 10.1007/s00345-015-1650-0

**Published:** 2015-08-04

**Authors:** Tristan Barrett, Andrew J. Patterson, Brendan C. Koo, Karan Wadhwa, Anne Y. Warren, Andrew Doble, Vincent J. Gnanapragasam, Christof Kastner, Ferdia A. Gallagher

**Affiliations:** Department of Radiology, Addenbrooke’s Hospital, Cambridge, CB2 0QQ UK; University of Cambridge, Cambridge, CB2 0QQ UK; Department of Urology, Addenbrooke’s Hospital, Cambridge, CB2 0QQ UK; Department of Histopathology, Addenbrooke’s Hospital, Cambridge, CB2 0QQ UK; Translational Prostate Cancer Group, Department of Oncology, Hutchinson-MRC Research Centre, University of Cambridge, Cambridge, UK

**Keywords:** Image-guided biopsy, Magnetic resonance imaging, Ultrasound, Transperineal, Prostate cancer

## Abstract

**Purpose:**

To compare histological outcomes in patients undergoing MRI–transrectal ultrasound fusion transperineal (MTTP) prostate biopsy and determine the incremental benefit of targeted cores.

**Methods:**

Seventy-six consecutive patients with 89 MRI-identified targets underwent MTTP biopsy. Separate targeted biopsies and background cores were obtained according to a standardized protocol. Target biopsies were considered of added diagnostic value if these cores showed a higher Gleason grade than non-targeted cores taken from the same sector (Group 1, *n* = 41). Conversely, where background cores demonstrated an equal or higher Gleason grade, target cores were considered to be non-beneficial (Group 2, *n* = 48).

**Results:**

There was no significant difference in age, PSA, prostate volume, time-to-biopsy, and number of cores obtained between the groups. A greater proportion of target cores were positive for cancer (158/228; 69.3 %) compared to background (344/1881; 18.38 %). The median target volume was 0.54 cm^3^ for Group 1 (range 0.09–2.79 cm^3^) and 1.65 cm^3^ for Group 2 (0.3–9.07 cm^3^), *p* < 0.001. The targets in Group 1 had statistically lower diameters for short and long axes, even after correction for gland size. The highest area under the receiver operating characteristic curve was demonstrated when a lesion cutoff value of 1.0 cm in short axis was applied, resulting in a sensitivity of 83.3 % and a specificity of 82.9 %.

**Conclusions:**

When a combined systematic and targeted transperineal prostate biopsy is performed, there is limited benefit in acquiring additional cores from larger-volume targets with a short axis diameter >1.0 cm.

## Introduction

Prostate cancer is unique among solid organ tumors in being predominantly diagnosed by an indirect, non-targeted biopsy method. However, transrectal ultrasound-guided (TRUS) biopsy is constrained by inherent sampling error and suboptimal detection efficiency, failing to detect up to 30 % of cancers [1, 2] and underestimating tumor aggressiveness in around one-third of cases [3]. There is now a substantial body of evidence validating multi-parametric magnetic resonance imaging (mp-MRI) as a means of detecting prostate tumors [4, 5]. Furthermore, MRI-guided targeting has been shown to significantly improve risk stratification by reducing sampling error [6]. As a result, some authors now recommend mp-MRI as a means of directing either initial or repeat biopsies of the prostate, following a previous negative TRUS [7, 8]. Increasingly, a transperineal (TP) approach to biopsy is being utilized as it offers several advantages over the transrectal route, including reduced rates of sepsis and an increased ability to sample tumors located in the anterior and apical regions of the prostate [9, 10].

MRI-targeted prostate biopsies have significantly higher rates of detection for clinically significant cancer, being associated with a higher percentage of positive cores and longer maximum cancer core length (MCCL) compared to systematic biopsies [11–13]. Targeted cores have also been shown to reduce the detection of incidental, clinically insignificant tumors [14, 15]. However, mp-MRI is known to have a false-negative rate, which can depend on the experience of the reporting radiologist and the threshold used to define a positive MRI [16]. Therefore, the background gland is often sampled in addition to targeted cores to ensure that, in the case of negative targets, MRI occult tumor is not present elsewhere. Recent recommendations for MRI-guided transperineal biopsy from the Ginsburg study group suggest that additional cores should be acquired from the same sector as the target cores as well as defined standard background sectors [17]. However, the acquisition of additional target cores to achieve the correct diagnosis needs to be balanced against limiting the overall number of biopsies taken, as well as the cost and the time required to undertake the MRI fusion biopsies. Taking more cores may bring an increased risk of morbidity [18]. Previous work suggests saturation TP biopsies performed prior to radical prostatectomy can make surgery more challenging and lead to increased complication rates [19]. Additionally, the need for a general anesthetic with a transperineal approach has an effect on both patients and provider capacities and is a barrier to its widespread use. Importantly, which MRI targets are more likely to benefit from a targeted biopsy approach and which may be easily biopsied without image fusion is still unknown.

The current requirement is therefore to acquire the minimum number of biopsy cores that will simultaneously allow the detection of the index lesion or lesions, while minimizing the risk of false-negative results. The aim of this study was to compare the characteristics of targets in patients undergoing standardized MRI-guided prostate biopsy and to identify when targeted cores are most beneficial in terms of cancer yield and grade stratification. Ultimately, the aim was to determine whether there are cases where there is no added benefit in targeting the biopsy.

## Methods

### Patient cohort

This retrospective review of outcomes was granted ethical approval by the local review committee, with the need for written informed consent waived. Seventy-six consecutive patients undergoing targeted MRI–TRUS fusion transperineal (MTTP) biopsy over an 18-month period from April 2013 to September 2014 were identified, meeting the inclusion criteria of a high-probability target lesion identified by the radiologist (see below) and tumor subsequently confirmed by the pathologist. Data were recorded according to the standards of reporting for MRI-targeted biopsy studies (START) of the prostate [20].

### Magnetic resonance imaging

MRI was performed on a 3.0-T DiscoveryMR750 (General Electric Healthcare, Waukesha, USA) with an 8-channel phased-array coil. The imaging protocol included axial *T*_1_-weighted spin echo images of the pelvis and high-resolution *T*_2_-weighted (*T*_2_*W*) fast-recovery fast spin-echo images centered on the prostate in the axial (slice thickness 3 mm), coronal, and sagittal planes. Axial diffusion-weighted imaging (DWI) was performed using a customized dual-spin echo-planar imaging pulse sequence (*b* values 50, 750, 1000, 1400 s/mm^2^). Apparent diffusion coefficient (ADC) maps were reconstructed using software programmed with MATLAB (MathWorks, Natick, MA).

### Image analysis

MRI images were interpreted by two attending uroradiologists with between 3- and 5-year experience in reading prostate MRIs. *T*_2_*W* and DWI images were evaluated using the prostate imaging-reporting and data system (PI-RADS) criteria [21]. The scoring system of MRI targets was based on a Likert scale: 1—significant cancer highly unlikely, 2—significant cancer unlikely, 3—intermediate probability, 4—significant cancer likely, and 5—significant cancer highly likely. Positive MRIs were defined as having high-probability targets with a score of 4 or 5. In cases of a positive MRI, additional targeted biopsy cores were obtained. Targets were prospectively drawn as a region-of-interest (ROI) contour and saved to the picture archiving and communication system (PACS).

### MRI–TRUS fusion transperineal (MTTP) biopsy

Transperineal biopsies were performed using the Biopsee^®^ MRI–TRUS fusion biopsy platform (Medcom, Darmstadt, Germany). Briefly, a transrectal ultrasound probe is inserted and mounted on a brachytherapy gantry with a template grid for transperineal needle placement in the prostate. MR images are co-registered to ultrasound images allowing prospectively determined targets to be outlined, along with whole-gland outlines. MTTP biopsies were performed according to the standards proposed by the Ginsburg study group, with 24–36 core biopsies obtained from standardized sectors, depending on prostate size, and additional biopsy cores taken from MRI-defined target(s) [17]. Three types of biopsy core were therefore obtained: (1) background sector biopsy cores (BSB; systematic cores taken from Ginsburg-defined sectors), (2) target biopsy cores (TB; cores taken from the target delineated on MRI), and (3) target sector biopsy cores (TSB; cores taken from a sector which contains a target). Biopsies were performed by attending urologists with the knowledge of the MRI result and using the pre-defined ROIs to guide biopsy. Targeted cores were obtained before the sector biopsies, with all target and sector cores placed into separate histology pots.

### Data collection

The following data were collected: indications for biopsy; patient demographics; biopsy core information; histopathology outcomes; prostate volume; target volume; axial prostate; and axial target dimensions. Information relating to target volume and gland volume is prospectively stored on the BiopSee^®^ MRI–TRUS fusion device, and these data were retrospectively collected for all cases. In addition to volume, the axial dimensions of the target and prostate were also recorded as they represent simple, reproducible metrics which can be acquired as part of routine clinical reporting to determine the suitability for biopsy. Axial dimensions for prostate were obtained from the axial *T*_2_-weighted MRI by measuring the largest transverse and antero-posterior diameters. The targets were also measured on the axial *T*_2_ images, by first identifying the maximum diameter which was determined to be the “long axis” of the target; the “short axis” was determined as the measurement perpendicular to this long axis diameter in the same axial plane.

**Fig. 1:**
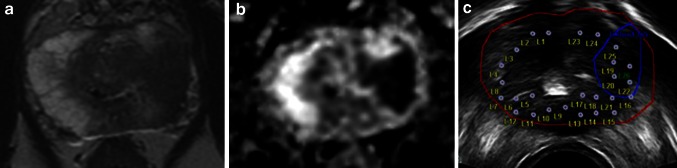
Target sector cores with higher Gleason grade than target cores. **a** Axial *T*2-weighted image shows a large lesion (volume 6.09 cm^3^) in the lateral and anterior left mid-peripheral zone, and **b** axial diffusion-weighted image shows marked restricted diffusion. **c** Peri-procedure axial US with target outline and biopsy plan. Both target cores demonstrated Gleason 4 + 3 disease, and background cores from the target sector demonstrated higher grade, Gleason 4 + 5 disease

Comparing the number of positive tumor cores acquired from targeted biopsies (TB) to the number of positive background cores taken from the same sector (TSB) provides a measure of the added benefit of undertaking these targeted biopsies. Targets were therefore retrospectively split into two groups for analysis: Group 1, where TB cores contained higher Gleason grade tumor than TSB cores; Group 2, where TSB cores contained an equal or higher Gleason grade than TB cores (Fig. 1).

### Statistical analysis

The distributions were assessed to establish whether they met normality assumptions. Statistical inferences were performed using the nonparametric Mann–Whitney *U* test to investigate the differences between groups in demographic and background information. Group differences in the planimetry-derived target volume metrics were also compared using this test. Fisher’s exact test was used to compare categorical Gleason data. Statistical significance was defined as a *p* value <0.05. Integrated areas under the receiver operating characteristic (ROC) curve were computed as determined by the relationship between specificity and sensitivity. The optimal cutoff for stratifying the groups was determined as the shortest Euclidean distance between 100 % specificity and sensitivity and the ROC curve. Pearson’s correlation was used to compare both total cores taken and cancer core involvement to tumor volume. All the statistical analyses were performed using the R programming language (version 3.1.1, The R foundation for statistical computing, Vienna, Austria) and the pROC package (version 1.7.3) [22].

## Results

Seventy-six patients with a median age of 68 years (range 53–76) and a median PSA of 8.9 ng/ml (range 0.8–53.2) met the inclusion criteria. A total of 89 high-probability MRI targets were identified (2 targets in 11 patients, 3 targets in 1 patient). The indication for biopsy and information on prior biopsy are listed in Table 1.

**Table 1:** Biopsy demographics

Indication for biopsy (*N* = 76)	
Raised PSA	18
Previous TRUS negative, rising PSA	15
Previous TRUS with suspicious changes	16
Active surveillance	26
BRCA-2 mutation	1
Number of previous biopsies (*N* = 76)	
0	13
1	47
2	9
≥3	7
Previous biopsy findings (*N* = 63)	
Benign	22
HGPIN	11
ASAP	4
Cancer	26
Gleason grade of previous positive biopsies (*N* = 26)	
3 + 3	18
3 + 4	5
4 + 3	3

*TRUS* transrectal ultrasound, *PSA* prostate-specific antigen, *HGPIN* high-grade prostatic intraepithelial neoplasia, *ASAP* atypical small acinar proliferation

Of 89 targets, 41 demonstrated higher Gleason grade in the target biopsy cores (Group 1), and 48/89 demonstrated equal or higher Gleason grade in the target sector biopsy cores (Group 2). There was no statistical difference between the two groups in terms of patient age, PSA, MRI score, prostate volume, time from MRI to biopsy, and total target or background biopsy cores obtained (Table 2). In 32/41 cases within Group 1, TSB were benign in comparison with the TB cores which showed tumor; in six cases, TSB cores showed Gleason 3 + 3 disease and TB cores showed ≥3 + 4 disease. In 30/48 cases in Group 2, the Gleason grade was equal in the TB and TSB cores; six cases had benign TB cores with positive corresponding TSB cores. Overall, a greater proportion of TB cores were positive for cancer (158/228; 69.3 %) compared to combined TSB and BSB cores (344/1881; 18.4 %) (Table 3).

**Table 2:** Patient demographics

	Overall	Group 1	Group 2	*p* value
Age (years)	53–76 (68)	53–75 (67)	53–76 (69)	0.368
PSA (ng/ml)	0.8–53.2 (8.9)	0.8–32 (8.9)	1.2–53.2 (9.9)	0.383
MRI score 4: *N*	33	18	15	0.273
MRI score 5: *N*	56	23	33	
Gland volume (cm^3^)	13.9–292.6 (43.2)	13.9–175.7 (48.8)	16.86–292.6 (40.4)	0.089
Time from MRI to biopsy (days)	12–90 (48.5)	12–90 (44)	17–85 (50.5)	0.285
Target cores: total (range; median)	228 (1–7; 2)	106 (2–6; 2)	122 (1–7; 2)	0.760
Total cores: total (range; median)	2109 (19–34; 28)	1189 (19–32; 28)	1297 (20–34; 28)	0.118

Range (median) listed, unless stated. Group 1: target biopsy cores show higher Gleason grade; Group 2: target sector biopsy cores show equal or higher Gleason grade

**Table 3:** Pathology outcomes

	Overall	Group 1	Group 2	*p* value
Target cores positive	158/228 (69.3 %)	76/106 (71.7 %)	82/122 (67.2 %)	0.476
Background cores positive	344/1881 (18.38 %)	101/876 (11.5 %)	243/1005 (24.2 %)	<0.001
Core mean length: range (median)	5.6–14.7 (10.35)	5.6–14.7 (10.15)	6.5–14.5 (10.5)	0.555
Final Gleason grade	(*n* = 76)	(*n* = 41)	(*n* = 48)	0.122
3 + 3	9 (11.8 %)	8 (9 %)	3 (3.4 %)	
3 + 4	32 (42.1 %)	21 (23.6 %)	19 (21.3 %)	
4 + 3	14 (18.4 %)	4 (4.5 %)	12 (13.4 %)	
8	5 (6.6 %)	3 (3.4 %)	3 (3.4 %)	
9	15 (19.7 %)	5 (5.6 %)	10 (11.2 %)	
10	1 (1.4 %)	0 (0 %)	1 (1.1 %)	

Group 1: target biopsy cores show higher Gleason grade; Group 2: target sector biopsy cores show equal or higher Gleason grade. Overall “final Gleason grade” listed as highest Gleason score per patient

**Fig. 2:**
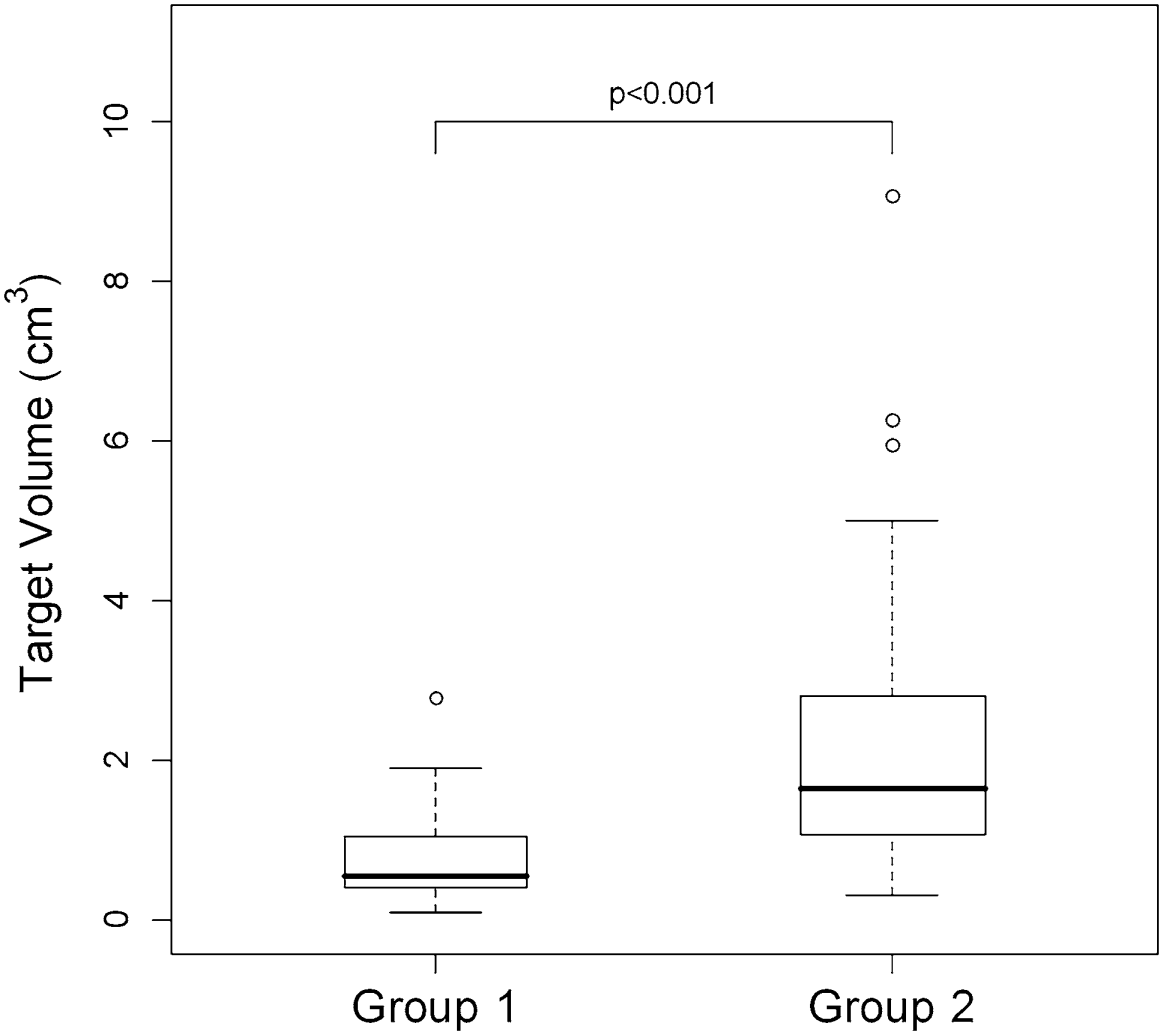
Box-and-whiskers plots comparing target volumes. The median target volume for Group 1 (0.54 cm^3^) was significantly lower than for Group 2 (1.65 cm^3^). The *upper* and *lower* limits of the *boxes* represent the 75th and 25th percentiles of the data, respectively, with the *line* in the *box* representing the median value; whiskers represent data within 1.5 times the interquartile range

The median overall target volume was 1.09 cm^3^ (mean 1.53, range 0.09–9.07 cm^3^). The median target volume for Group 1 was significantly lower at 0.54 cm^3^ [95 % confidence interval (CI) 0.41–1.04; range 0.09–2.79 cm^3^] compared to Group 2 with a median volume 1.65 cm^3^ (95 % CI 1.08–2.78, range 0.3–9.07 cm^3^), *p* < 0.001 (Fig. 2). This relationship held regardless of overall gland size, with results being similar when target volumes were expressed as a percentage of overall gland volume. Targets in Group 1 had a statistically significant lower longest and shortest axis diameter compared to Group 2, and this relationship was unchanged when diameter was expressed as a percentage of axial gland dimensions (Table 4).

**Table 4:** Lesion size parameters as a predictor of the incremental benefit of targeted cores

	Group 1	Group 2	*p* value	AUC	Cutoff	Spec (%)	Sens (%)
Target volume (cm^3^)	0.54 (0.41–1.04)	1.65 (1.08–2.78)	<0.001	0.837	0.815	85.4	68.3
Target volume as % of gland volume (%)	1.18 (0.83–1.93)	4.76 (1.90–7.09)	<0.001	0.837	2.348	70.8	87.8
Target longest axial diameter (cm)	1.37 (1.09–1.90)	2.04 (1.64–2.48)	<0.001	0.738	1.825	66.7	73.2
Target longest axial diameter as % of prostate TD (%)	27.8 (20.5–36.0)	42.3 (32.2–52.7)	<0.001	0.798	32.0	75.0	68.3
Target longest diameter as % of APD (%)	34.3 (27.8–47.7)	57.9 (40.9–70.2)	<0.001	0.784	53.3	58.3	87.8
Target shortest axial diameter (cm)	0.73 (0.65–0.98)	1.26 (1.08–1.71)	<0.001	0.886	0.995	83.3	82.9
Target shortest axial diameter as % of TD (%)	14.6 (12.0–17.1)	26.3 (22.4–32.5)	<0.001	0.927	19.1	89.6	85.4
Target shortest diameter as % of prostate APD (%)	18.5 (15.9–24.2)	36.9 (28.0–43.9)	<0.001	0.888	29.6	72.9	92.7

Group 1: target biopsy cores show higher Gleason grade; Group 2: target sector biopsy cores show equal or higher Gleason grade. Longest axial diameter relates to the maximal axial diameter of targets, and shortest diameter is the measurement perpendicular to this

*AUC* area under the curve, *TD* transverse diameter, *APD* antero-posterior diameter

**Fig. 3:**
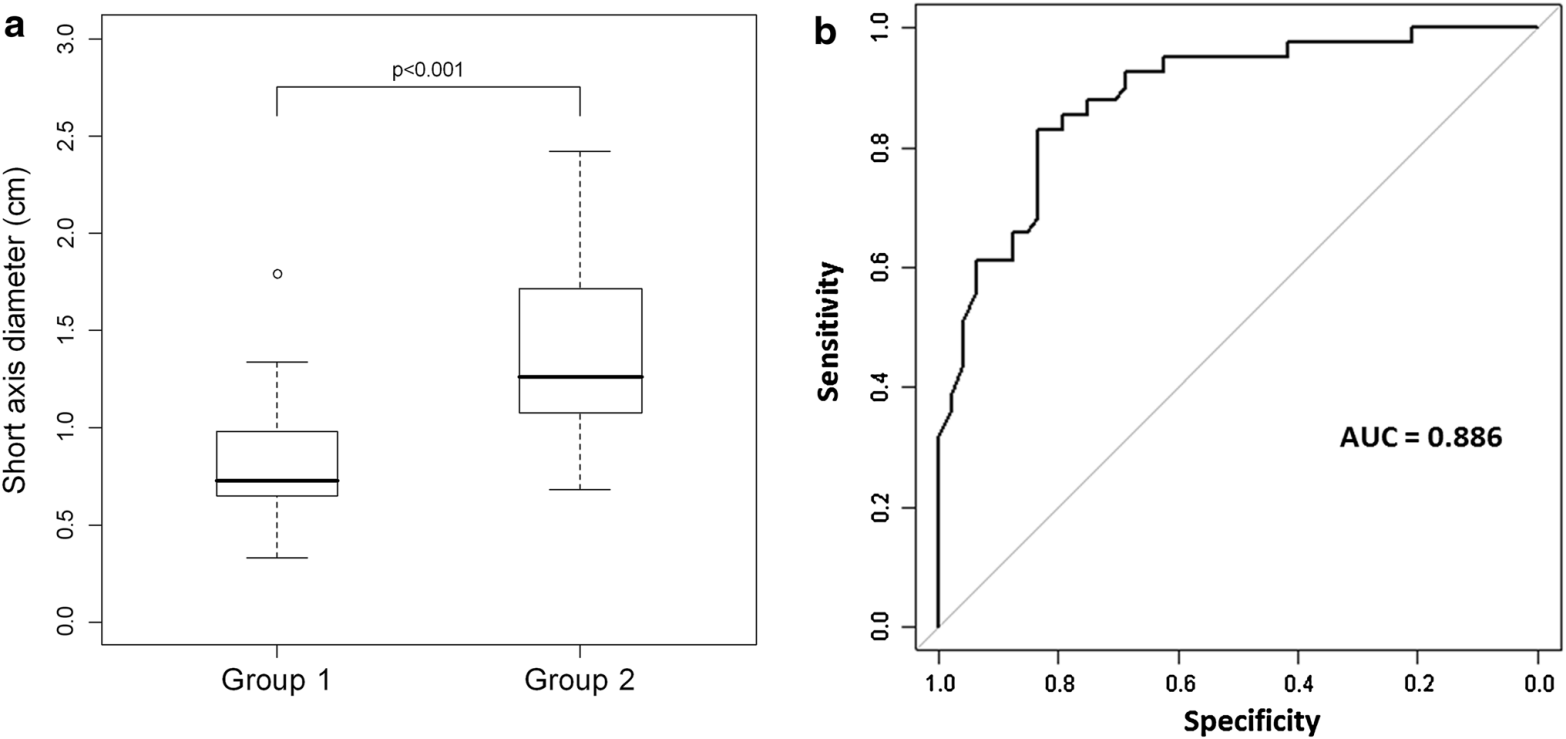
Comparing shortest axial diameter. The highest AUC for differentiating the two groups was demonstrated for the shortest axis diameter of the targets. **a** Box-and-whiskers plots and **b** receiver operating characteristics curves showing a significantly smaller measurement in Group 1, with a cutoff of 1.0 cm achieving a sensitivity of 83.3 and a specificity of 82.9 and a area under the curve (AUC) of 0.886

The area under the curve (AUC) of the receiver operating characteristic (ROC) curves ranged from 0.738 to 0.927 for all the variables assessed (Table 4). The highest AUC was demonstrated for the shortest axis measurement of targets, which demonstrated a sensitivity of 83.3 and a specificity of 82.9, at a cutoff value of 1.0 cm (Fig. 3).

There was no observable correlation between the number of target cores and the target volume (*r* = −0.055, *p* = 0.612). However, there was a positive correlation between the number of target sector cores acquired and the target volume (*r* = 0.322, *p* = 0.002); this is expected as the larger target will overlap with more sectors. For the 83 case with positive target cores, the average maximum cancer core length (MCCL) was 4.6 mm (range 0.5–15 mm) with a mean percentage core involvement of 34.1 % (range 1–95 %). There was a positive Pearson’s correlation between both MCCL and tumor volume (*r* = 0.693) and percentage core involvement and tumor volume (*r* = 0.554).

## Discussion

MRI is now established as the imaging modality of choice in the workup of organ-confined prostate cancer. MRI-guided targeted prostate biopsy is a logical next step to improve the accuracy and yield of diagnosis; however, this typically incurs additional costs in terms of the fusion software required and time taken to plan and perform procedures. Furthermore, although transperineal biopsies under local anesthetic have been described, a standardized validated technique is yet to be agreed, and reducing the number of cores may be the key to establishing this in select patients. This study has investigated the added value of MRI-targeted biopsies over non-targeted background cores and has demonstrated that there may be cases when there is no added benefit in undergoing a targeted biopsy in addition to the background template biopsies; in particular, it has addressed for the first time whether the size of the target may play a role in the sensitivity of MRI targeting.

This work has shown that there is a relationship between the size of a defined target and the additional value of targeted transperineal prostate biopsy for detecting the highest Gleason grade. Smaller targets were shown to upgrade tumors compared to background sampling, irrespective of whether absolute tumor size or tumor size relative to the gland was used. In routine practice, it is easier to define the two-dimensional measurements of a target rather than to determine volume, which requires multiple tumor outlines to be drawn and possibly the use of additional software. It is therefore clinically relevant to note that the short axis measurement of targets demonstrated the highest sensitivity and specificity, with a cutoff value of 1.0 cm. The improved performance of the short axis may reflect the fact that prostate tumors, and therefore targets, are often elliptical rather than circular, particularly in the anterior gland [23]. This is analogous to assessment of lymphadenopathy, where short axis measurements are the most sensitive indicator of tumor involvement [24]. Although further studies are required to validate this approach, this could provide a simple clinical metric for the determination of whether a lesion requires MRI targeting or not.

Target sector biopsy had a higher percentage of positive cores (69.3 %) compared to background cores (18.4 %). This was expected and is similar to previously published rates for positive target cores (range 46–67 %) and positive background cores (range 7.5–22 %) [13, 25, 26]. Group 2 had significantly more background cores positive (24.2 %) than Group 1 (11.5 %), which likely reflects the larger targets which involved more of the gland. Interestingly, there was no correlation between larger target size and an increased number of target cores obtained, which suggests that the clinicians performing the biopsy were attempting to limit the overall biopsy cores taken. There was a positive correlation between percentage core involvement by cancer and tumor volume, which serves as a quality control evaluation for correct needle placement.

**Fig. 4:**
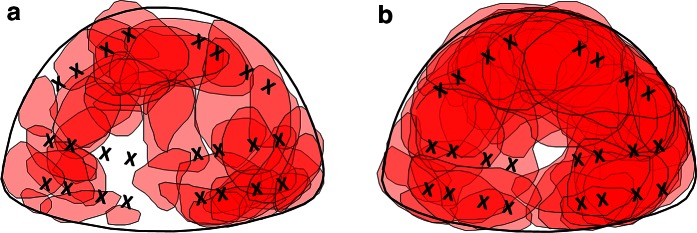
Location of targets. **a** Diagrammatic representation of all targets in Group 1 and **b** Group 2. The *X* on each image represents the location of the standard background sector biopsies

Our study has some limitations, including its retrospective nature. The cohort studied was predominantly a re-biopsy population (63/76 patients that had at least one prior biopsy). This may have introduced a selection bias toward smaller and more anterior tumors that would be missed by a TRUS approach. However, such a cohort represents the more typical patient population undergoing TP biopsy in current clinical practice. Location of a target was not assessed, and this may also be an important consideration in the utility of a targeted biopsy: For instance, if the target is remote from the location of the standard sectoral biopsy core, it is more likely to add value over the non-targeted biopsy (Fig. 4). However, this is difficult to objectively define, and target size will be an inherent confounding factor. Target cores were obtained before the background biopsies in this study. The initial biopsy could potentially reduce the volume of very small tumors or may affect the detection rate in subsequent cores, due to biopsy-induced hemorrhage. However, this effect would be similar between the two groups and therefore should not affect the outcome of this study.

Image-guided biopsy has been shown to improve cancer detection in patients with a previous negative prostate biopsy [27], as well as to reduce the detection rate of insignificant cancers [13]. As a result, targeted biopsy has the potential to reduce the number of cores required to diagnose significant prostate cancer, and theoretically, this could be used in isolation, without the need for a background biopsy [26, 28, 29]. While recent improvements in MRI have made it a sensitive technique for identifying tumor, the diagnostic challenge is to accurately characterize the index lesion while at the same time providing sufficient reassurance that there is no tumor when the MRI is negative, that is maximizing both sensitivity and negative predictive value (NPV). However, several published papers have shown that while MRI is sensitive, it does not yet have a sufficiently high NPV to provide the reassurance that there is no tumor elsewhere in the gland and therefore cannot currently be used to exclude the need for a biopsy of these areas. Consequently, the standard for prostate cancer detection remains a combination of targeted and systematic background biopsy cores [13, 17]. Although the current study does not directly define the added morbidity of additional cores acquired from larger-volume targets, previous work has shown that an increasing number of biopsy cores are associated with an elevated risk of morbidity, as well as complications at subsequent prostatectomy [18, 19]. Therefore, it is likely that the targeted biopsies acquired from these larger lesions provide little additional diagnostic information, but an increased risk of morbidity. Future prospective trials are required to evaluate this further.

In conclusion, the results of the study suggest that if a combined systematic and targeted transperineal prostate biopsy is performed, there is limited benefit in acquiring additional cores from larger-volume targets, when the short axis diameter is >1.0 cm.
